# Ferritin thresholds for cardiac and liver hemosiderosis in β-thalassemia patients: a diagnostic accuracy study

**DOI:** 10.1038/s41598-022-22234-9

**Published:** 2022-10-26

**Authors:** Hadi Darvishi-Khezri, Aily Aliasgharian, Mohammad Naderisorki, Mehrnoush Kosaryan, Mobin Ghazaiean, Hanie Fallah, Mohammad Zahedi, Hossein Karami

**Affiliations:** 1 Thalassemia Research Center (TRC), Hemoglobinopathy Institute, Mazandaran University of Medical Sciences, Sari, Iran; 2 Student Research Committee, Mazandaran University of Medical Sciences, Sari, Iran; 3 Department of Medical Biotechnology, Student Research Committee, School of Allied Medicine, Iran University of Medical Sciences, Tehran, Iran

**Keywords:** Ion channels, Metabolomics, Haematological diseases, Biochemistry, Diseases, Medical research, Risk factors

## Abstract

Ferritin is frequently used to screen some dire consequences of iron overload in β-thalassemia patients. The study aimed to define the best cutoff point of ferritin to screen for cardiac and liver hemosiderosis in these cases. This was a registry-based study on β-thalassemia patients living throughout Mazandaran province, Iran (*n* = 1959). In this diagnostic research, the index test was ferritin levels measured by a chemiluminescent immunoassay. As a reference test, T2*-weighted magnetic resonance imaging (T2*-weighted MRI) was applied to determine cardiac and liver hemosiderosis. A cutoff point of 2027 ng/mL for ferritin showed a sensitivity of 50%, specificity 77.4%, PPV 42.1%, and NPV 82.5% for cardiac hemosiderosis (area under curve [AUC] 0.66, 95% CI 0.60–0.71, adjusted odds ratio [OR] 2.05, 95% CI 1.05–4.01). At an optimum cutoff point of 1090 ng/mL, sensitivity 66.7%, specificity 68%, PPV 82.9%, and NPV 46.8% for liver hemosiderosis were estimated (AUC 0.68, 95% CI 0.63–0.73, adjusted OR 3.93, 95% CI 2.02–7.64. The likelihood of cardiac hemosiderosis serum ferritin levels below 2027 ng/mL is 17.5%. Moreover, 82.9% of β-thalassemia patients with serum ferritin levels above 1090 ng/mL may suffer from liver hemosiderosis, regardless of the grades.

## Introduction

β-thalassemia is a hereditary disease defined by a lack of β-chain globin synthesis^1^. Hemolysis, ineffective erythropoiesis, splenomegaly, and extramedullary hematopoiesis generate fatal anemia, ranging from non-transfusion-dependent thalassemia (NTDT) to transfusion-dependent thalassemia (TDT)^2,3^. An iron overload ensuing from iron hyper-absorption from the intestine, hemoglobin instability, and red-cell transfusion is well-documented as the most problematic issue, bringing several dire ramifications to these patients, such as cardiac and liver hemosiderosis^4,5^.

Since cardiac and liver hemosiderosis, especially the moderate-to-severe one, as the evident signs of iron overload, have been the most clinical concerns and also the primary causes of mortality in this population, β-thalassemia must be screened for these severe adverse complications^6,7^.Various indices and methods are utilized to assess iron levels in these patients, including serum markers of labile iron (ferritin and transferrin saturation), echocardiography, and magnetic resonance imaging (MRI)^8^. T2*-weighted magnetic resonance imaging (T2*-weighted MRI) is a trusted measurement estimating the levels of iron deposition in the heart and liver^9^. Besides a rather unavailability, MRI has been an expensive assay, rending introducing the ferritin test as an alternative test to screen organs involved by hemosiderosis in such an iron-overloaded state^10^. Although serum ferritin reflects iron stores, its values are unreliable in the case of some concomitant illnesses surging ferritin levels, including malignancies, infectious diseases, hepatitis, and critical and inflammatory conditions^11,12^.

The ferritin test is a non-specific test providing different sensitivities and specificities at dissimilar cutoff points for evaluating cardiac and liver hemosiderosis in multiple studies. Clinical sequelae, such as heart failure, cardiac dysrhythmia, and pulmonary hypertension from transfusional iron overload, still prevail in β-thalassemia patients^6,13,14^. In contrast, no consensus exists on the optimum cutoff of ferritin to screen for cardiac and liver hemosiderosis. Moreover, maintaining serum ferritin levels below the best cutoff may deter the incidence of cardiac and liver hemosiderosis in these cases. According to the literature, ferritin levels between 1500 and 2500 ng/mL have been proposed to predict cardiac hemosiderosis amid β-thalassemia cases^15–17^, while the values for apprising liver hemosiderosis were obtained from 500 to 1500 ng/mL^16–18^. Therefore, we decided to determine the cutoff point and diagnostic test values of the serum ferritin test to evaluate cardiac and liver hemosiderosis in this population.

## Materials and methods

### Ethical consideration

The study was approved by the Ethics Committee and Institutional Review Board (IRB) of Mazandaran University of Medical Sciences (MAZUMS), Sari, Iran (IR.MAZUMS.REC.1399.809). The university vouched for the study protocol, and all methods were performed in accordance with the relevant guidelines and regulations. After taking informed consent from the patients or their parents/guardians, patients' medical files were registered in the online database. The cases kept sure their information would be confidential and their identity will not reveal under any circumstances.

### Study design and patients

The current study was a diagnostic accuracy study performed cross-sectionally through data recorded (from 2018 to 2019) in the Mazandaran Thalassemia Registry (MTR) in which 14 users have registered information concerning β-thalassemia cases in 13 different cities across Mazandaran province. The study population included patients who had been entered with the definitive diagnosis of β-thalassemia in the registry located in Bu Ali Sina Hospital, Sari, Iran. The data comprised patients' demographic, clinical, laboratory, and therapeutic findings, which are available on our website of http://reg.mazums.ac.ir/login.aspx.

### Inclusion and exclusion criteria

The inclusion criterion was documented β-thalassemia cases, both NTDT and TDT. Alongside clinical manifestations, genetic tests and first hemoglobin electrophoresis contributed to confirming the diagnosis. The lack of ferritin test results and MRI data on the heart and liver was considered the exclusion criteria.

### Data collection

Variables included demographic data, such as age, sex, weight (kg), ferritin levels (ng/mL), parathyroid hormone (PTH) (pg/mL), 25-OHD3 1,25-dihydroxyvitamin D levels (vitamin D3, ng/mL), hemoglobin level (g/dL), history of diabetes mellitus, history of splenectomy, hepatomegaly, dependency on red-cell transfusion, use of hydroxyurea, deferiprone (DFP), deferoxamine (DFO), deferasirox (DFX), vitamin D3 supplementation, folic acid, estrogen, the severity of cardiac and liver hemosiderosis.

### Index test

To measure the serum ferritin levels, a chemiluminescent immunoassay was applied in our study (coefficient of variation [CV] 4.1 ng/mL, the limit of detection [LOD] 0.5 ng/mL). The last recorded values for each patient were entered in this work.

2.6. Reference test for diagnosing cardiac and liver hemosiderosis.

To determine the grade of iron overload, a T2* MRI was utilized for the heart and liver organs. The T2*-weighted MRI of the cardiac and liver was carried out employing a 1.5 Tesla scanner. Using T2* MRI, tissue iron was indirectly detected based on the duration of relaxation of iron deposited in the tissues^19^. Moreover, cardiac T2* MRI ≥ 20 s (sec), 14–19.99 s, 10–13.99 s, and < 10 s were categorized as normal, mild, moderate, and severe hemosiderosis, respectively^20^. In hepatic view, liver iron concentration (LIC) > 6.3 mg/gr/dry weight, 2.8–6.3 mg/gr/dry weight, 1.4–2.79 mg/gr/dry weight, and < 1.4 mg/gr/dry weight were also itemized as normal, mild, moderate and severe hemosiderosis, correspondingly^20^. All last registered MRI data were included in the study to consider the nearest values to the ferritin measures.

### Statistical analysis

Mean ± standard deviation or number (percentage) was used to present the results. We checked the normal distribution of ferritin according to the groups through a histogram plot and Shapiro–Wilk test. The independent student’s t- and chi-square tests were applied to compare the variables between two groups, cardiac/liver hemosiderosis, and non-cardiac/sliver hemosiderosis. A receiver operating characteristic (ROC) analysis was run using serum ferritin levels as an index test alongside any grade of cardiac/liver hemosiderosis and moderate-to-severe cardiac/liver hemosiderosis as the adverse events. We also made a composite test to check a possibility in the betterment of screening test values for liver and cardiac hemosiderosis, which was accompanying serum ferritin levels with weight (kg), age, PTH levels, and vitamin D3 levels; [ferritin + weight + age^2 + PTH + vitamin D3 levels]. The optimum cutoff points, sensitivity, specificity, positive predictive value (PPV), and negative predictive value (NPV) were estimated through the Youden method. After finding the best cutoff points of serum ferritin levels, the values were categorized and turned into dichotomous variables. Then, logistic regression was utilized to reckon the odds ratio (OR) of the adverse effects, with a 95% confidence interval (CI). Next, the impact of multiple potential confounders, including age, red-cell transfusion dependency, DFP, DFO, DFX, and hydroxyurea, were controlled to estimate an adjusted OR. In the final model, the multicollinearity between independent variables was assessed by the LMCOL command and VIF index (variance inflation factor). The goodness of the final model was also examined using the Homser-Lemeshow test. Ultimately, sub-group analyses were implemented based on splenectomized and non-splenectomized cases and iron chelators to define the best cutoff point for each sub-category. All statistical tests were executed by STATA version 13 (StataCorp, College Station, TX, USA) and MedCalc statistical software version 13. A P-value less than 0.05 was chosen as the threshold of statistical significance. As the null zone of the area under the ROC curve (AUC) is 0.5, encompassing this point by the estimated CIs reflects a statistical significance.

## Results

Of the 1959 registered thalassemia cases, 510 patients (26.03%) had ferritin data in the dataset. The mean of serum ferritin was 1847 ± 1822 ng/mL (median 1233 ng/mL). The basic characteristics of β-thalassemia patients according to cardiac and liver hemosiderosis have been shown in Table 1.

**Table 1:** Basic characteristics.

	Cardiac hemosiderosis		*P*-value	Liver hemosiderosis		*P*-value
	Yes (*n* = 105)	No (*n* = 269 )		Yes (*n* = 281)	No (*n* = 119)	
Age, year	31 ± 8	34 ± 8	0.005	32 ± 8	34 ± 9	0.08
Weight, kg	57 ± 8	59 ± 10	0.27	58 ± 10	59 ± 9	0.44
Gender, male/female	45/60	121/175	0.72	122/159	43/76	0.18
Transfusion dependent	103 (98.10)	245 (83.33)	** < 0.001**	244 (86.8)	103 (86.5)	0.81
DFP	68 (64.8)	74 (27.5)	** < 0.001**	112 (39.8)	29 (24.4)	**0.01**
DFO	80 (76.2)	149 (55.4)	** < 0.001**	179 (63.7)	49 (41.2)	** < 0.001**
DFX	17 (16.2)	90 (33.4)	**0.001**	70 (24.9)	37 (31.1)	0.05
Diabetes mellitus	15 (14.3)	26 (6.7)	0.08	28 (10)	13 (10.9)	0.90
Estrogen	3 (2.8)	4 (1.5)	0.37	4 (1.4)	3 (2.5)	0.36
Folic acid	83 (79)	223 (82.9)	0.35	219 (77.9)	86 (72.3)	0.31
Hemoglobin, gr/dL	8.83 ± 0.75	9.18 ± 5.05	0.51	9.17 ± 5.23	8.94 ± 1.06	0.94
Hepatomegaly	15 (14.3)	46 (17.1)	0.73	37 (13.2)	24 (20.2)	0.06
Hydroxyurea	14 (13.3)	77 (28.6)	**0.002**	63 (22.4)	28 (23.5)	0.41
Splenectomy	42 (40)	116 (43.1)	0.86	113 (40.2)	44 (37)	0.74
25-OHD3 level, ng/mL	34.56 ± 21.53	38.30 ± 21.54	0.16	35.58 ± 19.76	41.49 ± 24.73	0.02
Vitamin D supplementation	74 (70.5)	210 (78)	0.08	204 (72.6)	79(66.4)	0.69
25-OHD3 < 50 ng/mL	68 (64.8)	189 (70.3)	0.40	184 (65.5)	72 (60.5)	0.05

Significant values are in [bold].

Significance level was defined as a *P*-value lower than 0.05.

*DFP* deferiprone, *DFO* deferoxamine, *DFX* deferasirox, *25-OHD3* 1,25-dihydroxyvitamin D3.

### Ferritin thresholds for cardiac hemosiderosis

**Figure 1:**
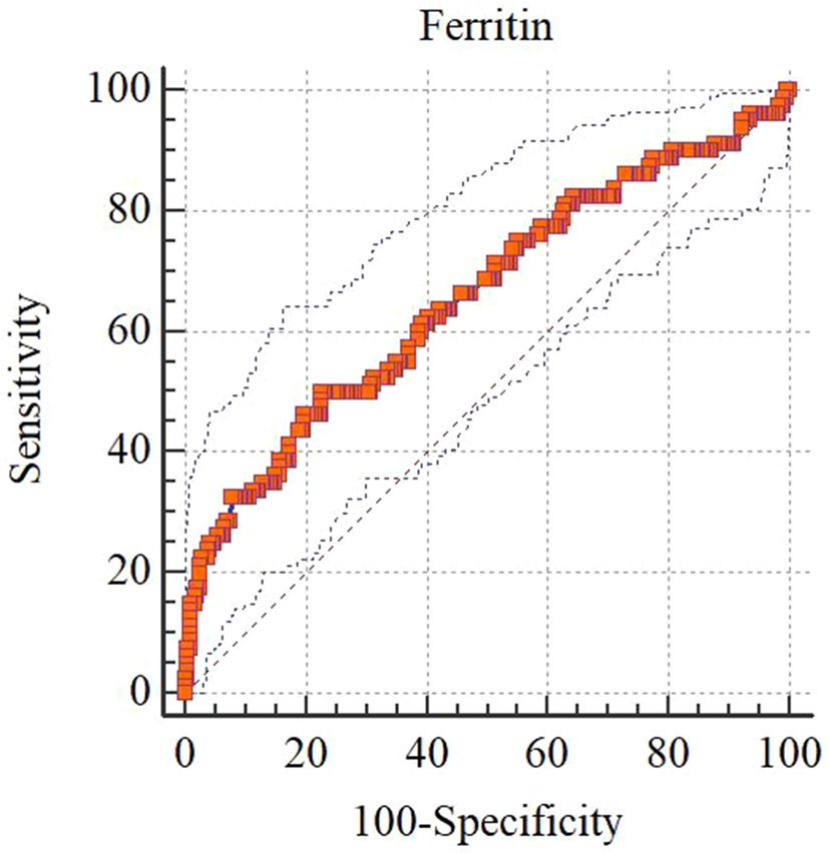
ROC curve of ferritin test for screening any grade of cardiac hemosiderosis (*n* = 374). Area under the ROC curve (AUC) 0.66 (95% CI 0.60–0.71), *P* < 0.001.

Serum ferritin level in cases with cardiac hemosiderosis was 2891 ± 2594 ng/mL, 1535 ± 1389 ng/mL in non-cardiac hemosiderosis (mean difference 1356 ng/mL; *P* < 0.001). A ferritin cutoff point of 2027 ng/mL showed a sensitivity of 50% (95% CI 38.6–61.4), specificity 77.4% (95% CI 71.6–82.5), PPV 42.1% (95% CI 32–52.7), and NPV 82.5% (95% CI 76.9–87.2) for cardiac hemosiderosis (AUC 0.66, 95% CI 0.60–0.71; *P* < 0.001). In cardiac hemosiderosis group, 53 cases (50%) had ferritin level more than 2027 ng/mL versus 208 cases (77.4%) in non-cardiac hemosiderosis. 50% of cases with cardiac hemosiderosis (52 cases) had serum ferritin levels greater than 2027 ng/mL versus 22.6% (61 cases) with serum ferritin levels > 2027 ng/mL (*P* < 0.001). The crude odds of cardiac hemosiderosis in cases with ferritin levels of more than 2027 ng/mL was 3.42 (95% CI 2.01–5.81) (adjusted OR 2.05, 95% CI 1.05–4.01; *P* = 0.03) (Table 2 and Fig. 1).

**Table 2:** Cutoff points and diagnostic values of ferritin test for any grade of cardiac hemosiderosis (*n* = 374).

Cutoff points	Sensitivity,%	95% CI	Specificity,%	95% CI	PPV,%	95% CI	NPV,%	95% CI
> 900	75.00	64.1–84.0	43.21	36.9–49.7	30.3	24.0–37.2	84.0	76.4–89.9
> 1000	71.25	60.0–80.8	46.09	39.7–52.6	30.3	23.8–37.4	83.0	75.5–88.9
> 1100	70.00	58.7–79.7	48.56	42.1–55.0	30.9	24.3–38.2	83.1	75.9–88.9
> 1200	66.25	54.8–76.4	52.67	46.2–59.1	31.5	24.6–39.2	82.6	75.7–88.2
> 1300	63.75	52.2–74.2	55.97	49.5–62.3	32.3	25.1–40.2	82.4	75.7–87.9
> 1400	62.50	51.0–73.1	59.67	53.2–65.9	33.8	26.2–42.0	82.9	76.4–88.1
> 1500	57.50	45.9–68.5	62.96	56.6–69.0	33.8	25.9–42.4	81.8	75.5–87.1
> 1600	55.00	43.5–66.2	64.20	57.8–70.2	33.6	25.6–42.4	81.2	75.0–86.5
> 1700	52.50	41.0–63.8	67.08	60.8–73.0	34.4	26.1–43.6	81.1	75.0–86.3
> 1800	52.50	41.0–63.8	68.72	62.5–74.5	35.6	27.0–44.9	81.5	75.5–86.5
> 1900	50.00	38.6–61.4	72.02	65.9–77.6	37.0	27.9–46.9	81.4	75.5–86.4
> 2000	50.00	38.6–61.4	76.54	70.7–81.7	41.2	31.3–51.7	82.3	76.7–87.0
** > 2027**	**50.00**	**38.6–61.4**	**77.37**	**71.6–82.5**	**42.1**	**32.0–52.7**	**82.5**	**76.9–87.2**
> 2200	46.25	35.0–57.8	77.78	72.0–82.8	40.7	30.5–51.5	81.5	75.9–86.2
> 2300	46.25	35.0–57.8	79.42	73.8–84.3	42.5	32.0–53.6	81.8	76.3–86.5
> 2400	43.75	32.7–55.3	80.66	75.1–85.4	42.7	31.8–54.1	81.3	75.8–86.0
> 2500	41.25	30.4–52.8	82.72	77.4–87.3	44.0	32.5–55.9	81.0	75.6–85.7

The best cutoff point (ng/mL) based on empirical cutoff point estimation (Youden method) was > 2027.

Furthermore, with a cutoff point of 2420 ng/mL, ferritin test demonstrated a sensitivity of 58.8% (95% CI 40.7–75.4), specificity 78.9% (95% CI 73.7–83.5), PPV 24.7% (95% CI 15.8–35.5), and NPV 94.2% (95% CI 90.5–96.8) for moderate-to-severe cardiac hemosiderosis (AUC 0.73, 95% CI 0.67–0.77; *P* < 0.001).

In the moderate-to-severe cardiac hemosiderosis group, ferritin levels of more than 2420 ng/mL were found in 27 cases (58.7%), compared to 69 cases (21%) in non-moderate-to-severe cardiac hemosiderosis cases. 41.3% of cases (19 cases) had ferritin levels over 2420 ng/mL, versus 79% (259 cases) in the non-moderate-to-severe cardiac hemosiderosis (P < 0.001). Ferritin level more than 2420 ng/mL represented a crude odds of 5.34, 95% CI 2.55–11.18 for moderate-to-severe cardiac hemosiderosis (adjusted OR 2.57, 95% CI 0.99–6.69; *P* = 0.05) (Supplementary Table 1 and Supplementary Fig. 1).

### Ferritin thresholds for liver hemosiderosis

**Figure 2:**
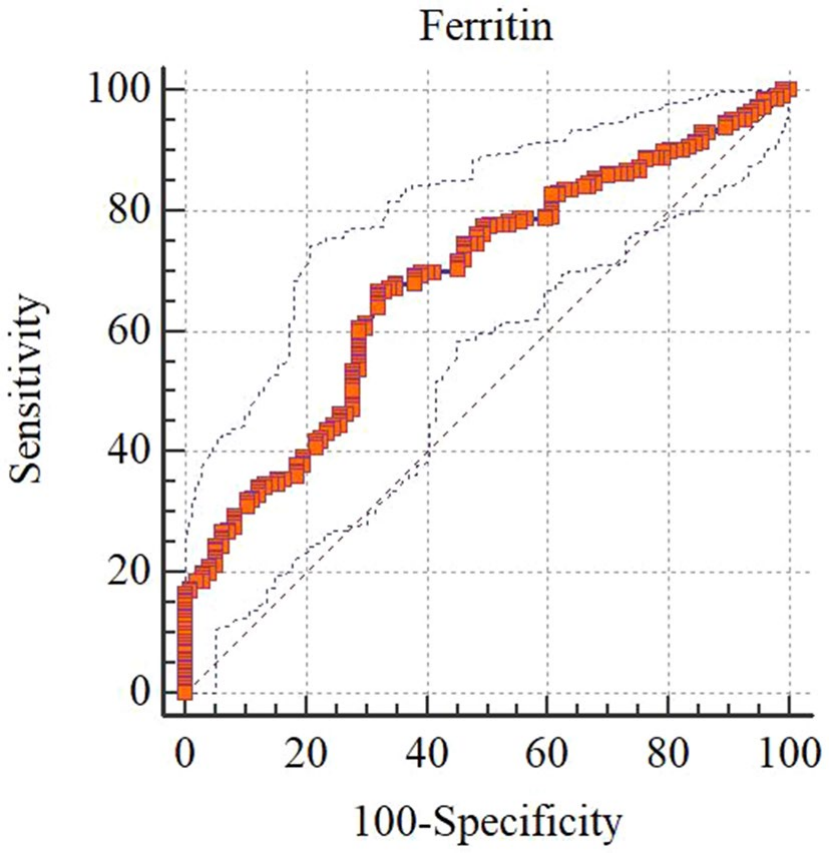
ROC curve of ferritin test for screening any grade of liver hemosiderosis (*n* = 400). Area under the ROC curve (AUC) 0.68 (95% CI 0.63–0.73), *P* < 0.001.

The mean of serum ferritin in cases with liver hemosiderosis was 2169 ± 1993 ng/mL, while the value in non-liver hemosiderosis was 1094 ± 885 ng/mL (mean difference 1075 ng/mL *P* < 0.001). At a cutoff point of serum ferritin level of 1090 ng/mL, the value reached a sensitivity of 66.7% (95% CI 60.1–72.8), providing a specificity of 68% (95% CI 57.8–77.1), PPV 82.9% (95% CI 76.6–88.1), and NPV 46.8% (95% CI 38.4–55.4) for screening liver hemosiderosis (AUC 0.68, 95% CI 0.63–0.73; *P* < 0.001). The proportion of cases with liver hemosiderosis and serum ferritin level higher than 1090 ng/mL was 66.5% (187 cases), compared to 31.9% (38 cases) in non-liver hemosiderosis. 33.5% of liver hemosiderosis cases (94 cases) had serum ferritin levels of 1090 ng/mL or less, compared to 68.1% (81 cases) in non-liver hemosiderosis (*P* < 0.001). Those who had ferritin levels over 1090 ng/mL provided crude odds of 4.26, 95% CI 2.56–7.08 for liver hemosiderosis (adjusted OR 3.93, 95% CI 2.02–7.64; *P* < 0.001) (Table 3 and Fig. 2).

**Table 3:** Cutoff points and diagnostic values of ferritin test for any grade of liver hemosiderosis (*n* = 400).

Cutoff points	Sensitivity,%	95% CI	Specificity,%	95% CI	PPV,%	95% CI	NPV,%	95% CIs
> 900	69.78	63.3–75.7	58.76	48.3–68.7	79.7	73.4–85.1	45.6	36.7–54.7
> 1000	68.00	61.5–74.0	64.95	54.6–74.4	81.8	75.5–87.1	46.7	38.0–55.4
** > 1090**	**66.67**	**60.1–72.8**	**68.04**	**57.8–77.1**	**82.9**	**76.6–88.1**	**46.8**	**38.4–55.4**
> 1200	61.33	54.6–67.7	70.10	60.0–79.0	82.6	76.0–88.1	43.9	35.9–52.1
> 1300	57.33	50.6–63.9	71.13	61.0–79.9	82.2	75.3–87.8	41.8	34.2–49.7
> 1400	53.33	46.6–60.0	72.16	62.1–80.8	81.6	74.4–87.5	40.0	32.7–47.7
> 1500	48.00	41.3–54.7	72.16	62.1–80.8	80.0	72.3–86.4	37.4	30.5–44.8
> 1600	46.22	39.6–53.0	73.20	63.2–81.7	80.0	72.1–86.5	37.0	30.1–44.2
> 1700	43.56	37.0–50.3	76.29	66.6–84.3	81.0	72.9–87.6	36.8	30.1–43.9
> 1800	42.22	35.7–49.0	77.32	67.7–85.2	81.2	72.9–87.8	36.6	30.0–43.6
> 1900	39.11	32.7–45.8	80.41	71.1–87.8	82.2	73.7–89.0	36.3	29.8–43.1
> 2000	35.56	29.3–42.2	83.51	74.6–90.3	83.3	74.4–90.2	35.8	29.6–42.5
> 2102	34.67	28.5–41.3	85.57	77.0–91.9	84.8	75.8–91.4	36.1	29.9–42.7
> 2200	34.22	28.0–40.8	86.60	78.2–92.7	85.6	76.6–92.1	36.2	30.0–42.8
> 2300	32.89	26.8–39.4	87.63	79.4–93.4	86.0	76.9–92.6	36.0	29.9–42.5
> 2400	31.56	25.5–38.1	89.69	81.9–94.9	87.7	78.5–93.9	36.1	30.0–42.5
> 2500	29.33	23.5–35.8	91.75	84.4–96.4	89.2	79.8–95.2	35.9	29.9–42.2

The best cutoff point (ng/mL) based on empirical cutoff point estimation (Youden method) was > 1090.

In cases with and without moderate-to-severe liver hemosiderosis, the mean of serum ferritin was 3179 ± 2732 ng/mL and 1245 ± 1095 ng/mL, respectively (*P* < 0.001). The cutoff-point of ferritin value for screening moderate-to-severe liver hemosiderosis was 1265 ng/mL, showing a sensitivity of 85% (95% CI 76.5–91.4) and a specificity 64.4% (95% CI 57.7–70.7) with a PPV of 51.8% (95% CI 43.9–59.7) and NPV of 90.5% (95% CI 84.8–94.6) (AUC 0.81, 95% CI 0.76–0.85; P < 0.001). In cases with and without moderate-to-severe liver hemosiderosis, 14.7% (19 cases) and 64.6% (175 cases) had serum ferritin levels of 1265 ng/mL or less, respectively. The proportion of cases with serum ferritin levels more than 1265 ng/mL was 85.3% (110 cases) in moderate-to-severe liver hemosiderosis cases, compared to 35.4% (96 cases) in non-moderate-to-severe liver hemosiderosis (*P* < 0.001). The crude odds of moderate-to-severe liver hemosiderosis in cases with ferritin levels higher than 1265 ng/mL was 10.26, 95% CI 5.55–18.95 (adjusted OR 9.66, 95% CI 4.11–22.68; *P* < 0.001) (Supplementary Table 2 and Supplementary Fig. 2).

### Sub-group analyses

The results of subgroup analysis according to splenectomy unveiled an optimum cutoff point of ferritin equal to 1367 ng/mL for cardiac hemosiderosis in the splenectomized cases, sensitivity 60% (95% CI 40.6–77.3), specificity 57.3% (95% CI 46.8–67.3), PPV 30.5% (95% CI 19.2–43.9), NPV 82.1% (95% CI 70.8%–90.4), AUC 0.59 (95% CI 0.48–0.69), OR 2.01 (95% CI 0.88–4.58). In this sub-group, the best ferritin cutoff point to screen moderate-to-severe cardiac hemosiderosis was defined at the value of 2022 ng/mL, sensitivity 62.5% (95% CI 35.4–84.8%), specificity 78.2% (95% CI 69.3–85.5), PPV 29.4% (95% CI 15.1–47.5), NPV 93.5% (95% CI 86.3–97.6), AUC 0.70 (95% CI 0.57–0.83), OR 5.97 (95% CI 2.03–17.5). To screen cardiac hemosiderosis, a best cutoff point anchored at the ferritin level of 2064 ng/mL, representing a sensitivity of 64.7% (95% CI 46.5–80.3), specificity 70.7% (95% CI 60.7–79.4), PPV 43.1% (95% CI 29.3–57.8), NPV 85.4% (95% CI 75.8–92.2), AUC 0.68 (95% CI 0.58–0.77), OR 4.43 (95% CI 1.96–10) amid the non-splenectomized cases, while the cutoff point for moderate-to-severe cardiac hemosiderosis set at 2447 ng/mL with sensitivity 72.7% (95% CI 39–94), specificity 73% (95% CI 64.2–80.6), PPV 19.5% (95% CI 8.82–34.9), NPV 96.7% (95% CI 90.8–99.3), AUC 0.73 (95% CI 0.58–0.87), OR 7.19 (95% CI 1.93–26.5).

The results as regards liver hemosiderosis introduced an optimum ferritin cutoff point of 1097 ng/mL amid the splenectomized cases, sensitivity 63.2% (95% CI 52.2–73.3), specificity 65.8% (95% CI 48.6–80.4), PPV 80.9% (95% CI 69.5–89.4), NPV 43.9% (95% CI 30.7–57.6), AUC 0.64 (95% CI 0.55–0.74), OR 3.31 (95% CI 1.5–7.29). The value for moderate-to-severe liver hemosiderosis stood at 1320 ng/mL, sensitivity 77.5% (95% CI 61.5–89.2), specificity 65.9% (95% CI 54.8–75.8), PPV 51.7% (95% CI 38.4–64.8), NPV 86.2% (95% CI 75.3–93.5), AUC 0.72 (95% CI 0.63–0.8), OR 6.65 (95% CI 2.83–15.6). In the non-splenectomized cases, the best ferritin cutoff point was obtained at 1234 ng/mL for screening liver hemosiderosis, sensitivity 71.6% (95% CI 61.4–80.4), specificity 65.8% (95% CI 48.6–80.4), PPV 84% (95% CI 74.1–91.2), NPV 48.1% (95% CI 34–62.4), AUC 0.69 (95% CI 0.60–0.78), OR 4.84 (95% CI 2.18–10.7), while the value for moderate-to-severe liver hemosiderosis set at 2325 ng/mL, representing a sensitivity of 71.1% (95% CI 55.7–83.6), specificity 85.2% (95% CI 76.1–91.9), PPV 71.1% (95% CI 55.7–83.6), NPV 85.2% (95% CI 76.1–91.9), AUC 0.78 (95% CI 0.70–0.86, OR 14.2 (95% CI 5.97–33.8).

Another sub-group analysis was also operated based on iron chelators. In DFP-treated cases, the best cutoff point was obtained at 2300 ng/mL for cardiac hemosiderosis, representing sensitivity 51.6% (95% CI 38.7–64.2), specificity 70.7% (95% CI 60.7–79.4), PPV 53.2% (95% CI 40.1–66), NPV 69.3% (95% CI 59.3–78.1), AUC 0.63 (95% CI 0.55–0.71), OR 5.16 (95% CI 1.97–13.47). In this patients, the value for moderate-to-severe cardiac hemosiderosis was estimated at 2700 ng/mL, sensitivity 60% (95% CI 40.6–77.3), specificity 77.4% (95% CI 69.4–84.2), PPV 37.5% (95% CI 24–52.6), NPV 89.6% (95% CI 82.5–94.5), AUC 0.70 (95% CI 0.62–0.77), OR 3.69 (95% CI 1.47–9.27). In this sub-group, 1056 ng/mL was the best cutoff point of ferritin for liver hemosiderosis, which estimated a sensitivity of 75.2% (95% CI 66.5–82.6), specificity 73.2% (95% CI 57.1–85.8), PPV 89.2% (95% CI 81.5–94.5), NPV 50% (95% CI 36.8–63.2), AUC 0.78 (95% CI 0.70–0.84), OR 8.79 (95% CI 3.19–24.2). For moderate-to-severe liver hemosiderosis, the value stood at 1265 ng/mL, sensitivity 90.6% (95% CI 80.7–96.5), specificity 62.2% (95% CI 51.9–71.8), PPV 61.1% (95% CI 50.5–70.9), NPV 91% (95% CI 81.5–96.6), AUC 0.83 (95% CI 0.76–0.88), OR 17.6 (95% CI 4.96–62.47).

In DFO-treated cases, 2300 ng/mL was the optimum cutoff point of ferritin for cardiac hemosiderosis, which provided a sensitivity of 48.7% (95% CI 37–60.4), specificity 72.4% (95% CI 65–78.9), PPV 44% (95% CI 33.2–55.3), NPV 75.9% (95% CI 68.6–82.3), AUC 0.63 (95% CI 0.56–0.69), OR 5.20 (95% CI 2.43–11.15). For moderate-to-severe cardiac hemosiderosis, the value was computed at 2700 ng/mL, sensitivity 52.9% (95% CI 35.1–70.2), specificity 78.8% (95% CI 72.6–84.1), PPV 28.6% (95% CI 17.9–41.3), NPV 91.3% (95% CI 86.2–94.9), AUC 0.69 (95% CI 0.62–0.74), OR 3.16 (95% CI 1.40–7.12). In this sub-group, the optimum ferritin cutoff point was calculated at 1056 ng/mL for screening liver hemosiderosis, sensitivity 73.1% (95% CI 66–79.4), specificity 61.9% (95% CI 48.8–73.9), PPV 84.7% (95% CI 78.1–90), NPV 44.3% (95% CI 33.7–55.3), AUC 0.69 (95% CI 0.63–0.75), OR 3.42 (95% CI 1.64–7.11). The value reached 1265 ng/mL for moderate-to-severe liver hemosiderosis, providing sensitivity 89.7% (95% CI 81.3–95.2), specificity 58.2% (95% CI 50.1–66), PPV 54.2% (95% CI 45.7–62.5), NPV 91.1% (95% CI 83.8–95.8), AUC 0.81 (95% CI 0.76–0.86), OR 11.02 (95% CI 4.42–27.46).

In DFX-treated cases, ferritin's best cutoff point value for cardiac hemosiderosis stood at 2027 ng/mL. The values of sensitivity, specificity, PPV, NPV, AUC, OR were estimated at 50% (95% CI 38/6–61/4), 76.3% (95% CI 70.3–81.7), 42.6% (95% CI 32.4–53.2), 81.3% (95% CI 75.4–86.3), 0.65 (95% CI 0.59–0.70) and 1.5 (95% CI.28–7.93), respectively. For moderate-to-severe cardiac hemosiderosis, the value reached 2420 ng/mL, generating a sensitivity of 58.8% (95% CI 40.7–75.4), specificity 77.7% (95% CI 72.3–82.5), PPV 24.7% (95% CI 15.8–35.5), NPV 93.8% (95% CI 89.9–96.6), AUC 0.72 (95% CI 0.66–0.77). In this sub-group, the cutoff point of ferritin for liver hemosiderosis was found at 1056 ng/mL, sensitivity 68.3% (95% CI 61.7–74.5), specificity 65.2% (95% CI 54.3–75), PPV 82.8% (95% CI 76.5–88), NPV 45.7% (95% CI 36.8–54.7), AUC 0.68 (95% CI 0.62–0.73), OR 2.97 (95% CI 1.23–7.16). The cutoff point stood at 1265 ng/mL for screening moderate-to-severe liver hemosiderosis, sensitivity 86.6% (95% CI 78.2–92.7), specificity 62.4% (95% CI 55.5–69), PPV 51.5% (95% CI 43.6–59.4), NPV 91% (95% CI 85.1–95.1), AUC 0.81 (95% CI 0.76–0.85), OR 4.4 (95% CI 1.38–13.99).

### The composite test

Considering the composite test, the ferritin sensitivity for screening moderate-to-severe cardiac hemosiderosis rose by 20.1%, reaching 78.9% (95% CI 54.4–93.9) at the optimum cutoff of 3397 ng/mL. Nevertheless, the other diagnostic values did not change compared to the ferritin test alone. The composite test revealed a specificity 74.5% (95% CI 67.1–81.1), PPV 26.8% (95% CI 15.8–40.3) and NPV 96.8% (95% CI 91.9–99.1), AUC 0.77 (95% CI 0.66–0.87) OR 11 (95% CI 3.6–33.2).

## Discussion

The most prevalent cause of mortality in patients with ∝ -thalassemia is iron-induced cardiomyopathy^21^, particularly in cases with chronic red-cell transfusion^22^. Cardiac dysfunction and heart failure are generally due to iron overloading in this population. Iron excess in severe thalassemia is caused by increased gastrointestinal absorption and constant red-cell transfusion^4^. The ferritin test seems simple and non-invasive to screen iron surplus and its adverse consequences^23–25^. Optimum cutoff points of the ferritin test to screen cardiac hemosiderosis (2027 ng/mL) and moderate-to-severe cardiac hemosiderosis (2420 ng/mL) showed a feeblish sensitivity and specificity.

Nevertheless, NPVs for both conditions were acceptable, 82.5% for cardiac hemosiderosis and 94.2% for moderate-to-severe cardiac hemosiderosis. Based on the current study, serum ferritin levels of more than 2420 ng/mL can pose β-thalassemia cases at risk of moderate-to-severe cardiac hemosiderosis, almost twofold. As is presented, the estimated point of serum ferritin for myocardial hemosiderosis was virtually half of the value calculated for liver hemosiderosis, reflecting that myocardial hemosiderosis as a late onset complication may happen in a more severe iron-overloaded status compared to liver hemosiderosis^16^.

Maintaining serum ferritin concentrations under 2500 ng/mL alongside iron chelators therapy can improve the survival rate without cardiac disease for TDT patients^26^. It was reported that patients with serum ferritin levels < 2500 ng/mL had an estimated cardiac disease-free survival of 91% after 15 years, while patients with serum ferritin levels > 2500 ng/mL witnessed a cardiac disease-free survival of less than 20% after 15 years^26^.

Spleen and liver hemosiderosis may correlate together and also with serum ferritin levels in conformity with a recent study^27^. In addition, an elevated risk of cardiac hemosiderosis and a liver enlargement may rise in patients who underwent splenectomy. We, therefore, performed a sub-group analysis based on splenectomy. Nonetheless, no evident change in the diagnostic values was observed. To be more exact, In the splenectomized cases, the optimum cutoff point for screening moderate-to-severe cardiac hemosiderosis saw a drop of around 400, reaching 2022 ng/mL. However, the value did not change in the non-splenectomized ones and remained around 2400.

The results from the sub-group analysis of the iron chelators used by the patients disclosed that in the cases under treatment with DFP and DFO, the optimum cutoff point of ferritin to screen cardiac hemosiderosis experienced an increase of nearly 300 ng/mL. This change in the ferritin threshold for screening cardiac hemosiderosis emphasizes the cardioprotective effect of DFP and DFO in iron overload conditions. Notwithstanding, to screen cardiac hemosiderosis, the value did not change for those who received DFX. Based on this evidence, cardiac hemosiderosis may be implemented at a higher serum ferritin level in patients who use DFP and DFO. Some studies have previously revealed that DFP has a superior performance to other iron chelators^28^ or in combination with DFO compared to DFO alone^29^ to deter developing cardiac hemosiderosis in this population. In addition, the cutoff point stood unchanged for screening liver hemosiderosis among patients under monotherapy with DFP, DFO, and DFX.

Our results suggest that the ferritin test would be more merit for screening for liver hemosiderosis than cardiac hemosiderosis in β-thalassemia patients. A serum ferritin level of 1090 ng/mL and more for screening liver hemosiderosis revealed the best PPV of 82.9%. Furthermore, the β-thalassemia cases with serum ferritin levels higher than this threshold had a nearly threefold raised risk of liver hemosiderosis. An acceptable sensitivity of 85% and NPV of 90.5% were obtained at the optimum cutoff point of ferritin for screening moderate-to-severe liver hemosiderosis (1265 ng/mL). The surge in ferritin levels above this threshold can ramp up the risk of moderate-to-severe liver hemosiderosis to approximately ninefold in these cases.

In light of the data presented, to screen for moderate-to-severe cardiac hemosiderosis, considering weight, age, PTH, and vitamin D3 levels alongside a ferritin test can boost the sensitivity value of ferritin by near 20% (optimum cutoff 3397). As the composite test failed to improve diagnostic values for detecting any grade of cardiac and liver hemosiderosis, this approach would be recommended merely for screening moderate-to-severe cardiac hemosiderosis. Intriguingly, a recent aged-matched case–control study on 51 non-hepatitis ∝-thalassemia major patients disclosed that the hepcidin/ferritin ratio could be a more appropriate tool to predict iron overload-induced liver fibrosis and discriminate its stages with acceptable sensitivity and specificity than the hepcidin and ferritin alone. The sensitivity and specificity were computed at 100%, while the estimated values to differentiate liver fibrosis stages were 90% and 89.5%, respectively^30^. Determining the diagnostic values of the hepcidin/ferritin ratio index to appraise cardiac hemosiderosis needs to be encouraged to elucidate its diagnostic application and usefulness in these cases.

A small retrospective study (*n* = 32)^31^ with a long follow-up time (median 13.6 years) conducted on β-thalassemia major concluded that maintaining serum ferritin levels below 1500 μg/L can significantly keep clinical complications arising from iron surplus at bay in this population (low risk). If these cases experience an increase in serum ferritin levels over 2500 μg/L, the likelihood of contracting complications will surge significantly (high risk)^31^. In that study, cases with low LIC (7 mg/g dry weight) determined by a liver biopsy tissue and the mean ferritin of 1500 mg/L witnessed no deaths or cardiac events (*n* = 8) over the study' timeframe. Importantly, that study propounded that minor oscillation in serum ferritin levels alongside keeping that under 1500 μg/L would be efficacious in preventing complications in these patients.

It has been recommended that serum ferritin levels of about 450 ng/mL can be assumed as a cutoff point to apprise liver hemosiderosis in NTDT cases. In this cutoff point, a sensitivity of 0.78% and specificity of 0.82% were estimated^18^. According to data extracted from another study^15^, raising serum ferritin over 2000 ng/mL is associated with nearly six times raised risk of myocardium hemosiderosis in ∝ -thalassemia, OR 7.38 (95% CI 2.04–26.74). It is propounded that ferritin levels are a more accurate marker for TDT cases than NTDT patients because of the underestimation of its level in patients with TDT. Therefore, initiation of chelation therapy of NTDT patients should be regarded in lower ferritin levels^25,32^.

Data as regards a diagnostic study^17^ depicted a better sensitivity and specificity of the ferritin test for screening cardiac hemosiderosis than liver hemosiderosis measured by MRI (*n* = 405). Ferritin cutoff points for cardiac and liver hemosiderosis were estimated at 2485 ng/mL (sensitivity 95.2% and specificity 73.4%) and at 1713 ng/mL (sensitivity 71% and specificity 76.9%), correspondingly. The diagnostic values computed for assessing cardiac, and liver hemosiderosis experienced no conspicuous changes when TDT cases were merely included. To be precise, the cutoff point of ferritin for screening cardiac hemosiderosis stood at 2485 ng/mL (sensitivity 94.4% and specificity 68.6%), while the value for liver hemosiderosis was 2071 ng/mL (sensitivity 63.2% and specificity 79.1%). In NTDT, the values of sensitivity and specificity calculated for screening cardiac hemosiderosis rose to 100% and 88.9% (cutoff point 3278 ng/mL), whereas the value for screening liver hemosiderosis reached 41.4% and 92.9% (cutoff point 1710 ng/mL), correspondingly^17^.

A ferritin is an intracellular form of iron storage, plays a primary role in the availability of serum iron. This metalloprotein is a general indication of the total iron storage^33,34^. It seems that ferritin levels alone would not be utterly adequate for screening cardiac and liver hemosiderosis because they may demonstrate considerable variations due to inflammation and infection^16^. Because of the impaction of ferritin levels by various conditions, measuring transferrin receptor levels could be an advantageous index to rule out inflammatory causes^25,32^. Therefore, exploring composite index or new simple markers are required.

Additionally, it should be noted that ferritin levels in each patient may see oscillations several times towards the upper threshold values, not following a linear trend over time^31^. These fluctuations and the frequency of crossing these cutoff points might escalate the risk of cardiac and liver hemosiderosis in this population, even if their serum ferritin levels remain under the thresholds. There have been examples of heavy hemosiderosis where the serum ferritin levels were disproportionately low^35^. However, conducting well-designed and robust studies is necessary to confirm this view.

## Conclusion

Respectively, the likelihood of cardiac hemosiderosis at any intensity and moderate-to-severe cardiac hemosiderosis in β-thalassemia patients with serum ferritin levels of less than 2027 ng/mL and 2420 ng/mL are 17.5% and 5.8%. Moreover, 82.9% of ∝-thalassemia patients with serum ferritin levels higher than 1090 ng/mL may suffer from any grade of liver hemosiderosis. The chance of moderate-to-severe liver hemosiderosis in β-thalassemia patients with serum ferritin levels of less than 1265 ng/mL is 9.5%. Based on our study, serum ferritin levels greater than 2027 ng/mL can be alarming for cardiac hemosiderosis in β-thalassemia patients. For liver hemosiderosis, serum ferritin levels can be avoided above 1090 ng/mL. Ferritin showed more value for screening liver hemosiderosis than cardiac hemosiderosis. Our study failed to estimate the median time between the ferritin test and cardiac and liver T2* MRI. Although the index and reference tests were not necessarily performed simultaneously, we considered the last registered data for both tests to diminish the risk of bias. Ultimately, the estimations of correlation between LIC, cardiac and liver T2* MRI with serum ferritin levels were not executed as the related data was not recorded in the registry.

## Supplementary Information

Supplementary Information.

## Data Availability

The data supporting this study's findings are available from the corresponding author upon reasonable request.
