# Supplementary material for: Ferritin thresholds for cardiac and liver hemosiderosis in β-thalassemia patients: a diagnostic accuracy study
Source: Sci Rep. 2022 Oct 26;12:17996. doi: 10.1038/s41598-022-22234-9 (PMC9606378; doi:10.1038/s41598-022-22234-9)
Supplement: Supplementary file 1 — Supplementary Information. [file 41598_2022_22234_MOESM1_ESM.docx]

**Supplementary Table 1.** Cutoff points and diagnostic values of ferritin test for moderate-to-severe cardiac hemosiderosis (n= 374)

| **Cutoff points** | **Sensitivity,%** | **95% CI** | **Specificity,%** | **95% CI** | **PPV,%** | **95% CI** | **NPV,%** | **95% CI** |
| --- | --- | --- | --- | --- | --- | --- | --- | --- |
| >900 | 79.41 | 62.1 - 91.3 | 40.83 | 35.1 - 46.7 | 13.6 | 9.2 - 19.2 | 94.4 | 88.8 - 97.7 |
| >1000 | 76.47 | 58.8 - 89.3 | 43.94 | 38.1 - 49.9 | 13.8 | 9.2 - 19.6 | 94.1 | 88.7 - 97.4 |
| >1100 | 76.47 | 58.8 - 89.3 | 46.37 | 40.5 - 52.3 | 14.4 | 9.6 - 20.3 | 94.4 | 89.2 - 97.5 |
| >1200 | 73.53 | 55.6 - 87.1 | 50.52 | 44.6 - 56.4 | 14.9 | 9.9 - 21.2 | 94.2 | 89.3 - 97.3 |
| >1300 | 73.53 | 55.6 - 87.1 | 53.98 | 48.0 - 59.8 | 15.8 | 10.5 - 22.5 | 94.5 | 89.9 - 97.5 |
| >1400 | 70.59 | 52.5 - 84.9 | 57.09 | 51.2 - 62.9 | 16.2 | 10.7 - 23.2 | 94.3 | 89.7 - 97.2 |
| >1500 | 70.59 | 52.5 - 84.9 | 61.25 | 55.4 - 66.9 | 17.6 | 11.6 - 25.1 | 94.7 | 90.4 - 97.4 |
| >1600 | 67.65 | 49.5 - 82.6 | 62.63 | 56.8 - 68.2 | 17.6 | 11.5 - 25.2 | 94.3 | 90.0 - 97.1 |
| >1700 | 64.71 | 46.5 - 80.3 | 65.40 | 59.6 - 70.9 | 18.0 | 11.7 - 26.0 | 94.0 | 89.8 - 96.9 |
| >1800 | 64.71 | 46.5 - 80.3 | 66.78 | 61.0 - 72.2 | 18.6 | 12.1 - 26.9 | 94.1 | 90.0 - 96.9 |
| >1900 | 61.76 | 43.6 - 77.8 | 69.90 | 64.2 - 75.1 | 19.4 | 12.5 - 28.2 | 94.0 | 89.9 - 96.7 |
| >2000 | 61.76 | 43.6 - 77.8 | 73.70 | 68.2 - 78.7 | 21.6 | 13.9 - 31.2 | 94.2 | 90.4 - 96.9 |
| >2102 | 58.82 | 40.7 - 75.4 | 74.74 | 69.3 - 79.6 | 21.5 | 13.7 - 31.2 | 93.9 | 90.0 - 96.6 |
| >2200 | 58.82 | 40.7 - 75.4 | 75.43 | 70.1 - 80.3 | 22.0 | 14.0 - 31.9 | 94.0 | 90.1 - 96.7 |
| >2300 | 58.82 | 40.7 - 75.4 | 76.82 | 71.5 - 81.6 | 23.0 | 14.6 - 33.2 | 94.1 | 90.2 - 96.7 |
| >2400 | 58.82 | 40.7 - 75.4 | 78.55 | 73.4 - 83.1 | 24.4 | 15.6 - 35.1 | 94.2 | 90.4 - 96.8 |
| **>2420** | **58.82** | **40.7 - 75.4** | **78.89** | **73.7 - 83.5** | **24.7** | **15.8 - 35.5** | **94.2** | **90.5 - 96.8** |
| >2500 | 55.88 | 37.9 - 72.8 | 80.62 | 75.6 - 85.0 | 25.3 | 16.0 - 36.7 | 94.0 | 90.2 - 96.6 |

The best cutoff point (ng/mL) based on empirical cutoff point estimation was (Youden method) >2420.

 **Supplementary Figure1.** ROC curve of ferritin test for screening moderate-to-severe cardiac hemosiderosis (n= 374). Area under the ROC curve (AUC) 0.73 (95% CI 0.67 – 0.77), P<0.001.

**Supplementary Table 2.** Cutoff points and diagnostic values of ferritin test for moderate-to-severe liver hemosiderosis (n= 400)

| **Cutoff points** | **Sensitivity,%** | **95% CI** | **Specificity,%** | **95% CI** | **PPV,%** | **95% CI** | **NPV,%** | **95% CI** |
| --- | --- | --- | --- | --- | --- | --- | --- | --- |
| >900 | 89.00 | 81.2 - 94.4 | 51.35 | 44.6 - 58.1 | 45.2 | 38.1 - 52.4 | 91.2 | 84.8 - 95.5 |
| >1000 | 88.00 | 80.0 - 93.6 | 55.41 | 48.6 - 62.1 | 47.1 | 39.7 - 54.5 | 91.1 | 85.0 - 95.3 |
| >1100 | 87.00 | 78.8 - 92.9 | 58.11 | 51.3 - 64.7 | 48.3 | 40.8 - 55.9 | 90.8 | 84.9 - 95.0 |
| >1200 | 85.00 | 76.5 - 91.4 | 63.06 | 56.3 - 69.4 | 50.9 | 43.1 - 58.7 | 90.3 | 84.5 - 94.5 |
| **>1265** | **85.00** | **76.5 - 91.4** | **64.41** | **57.7 - 70.7** | **51.8** | **43.9 - 59.7** | **90.5** | **84.8 - 94.6** |
| >1300 | 82.00 | 73.1 - 89.0 | 66.22 | 59.6 - 72.4 | 52.2 | 44.1 - 60.3 | 89.1 | 83.3 - 93.4 |
| >1400 | 77.00 | 67.5 - 84.8 | 68.47 | 61.9 - 74.5 | 52.4 | 44.0 - 60.7 | 86.9 | 80.9 - 91.5 |
| >1500 | 73.00 | 63.2 - 81.4 | 72.07 | 65.7 - 77.9 | 54.1 | 45.3 - 62.7 | 85.6 | 79.7 - 90.3 |
| >1600 | 69.00 | 59.0 - 77.9 | 72.52 | 66.1 - 78.3 | 53.1 | 44.1 - 61.9 | 83.9 | 77.9 - 88.8 |
| >1700 | 67.00 | 56.9 - 76.1 | 75.68 | 69.5 - 81.2 | 55.4 | 46.1 - 64.4 | 83.6 | 77.7 - 88.4 |
| >1800 | 65.00 | 54.8 - 74.3 | 76.58 | 70.4 - 82.0 | 55.6 | 46.1 - 64.7 | 82.9 | 77.1 - 87.8 |
| >1900 | 60.00 | 49.7 - 69.7 | 78.83 | 72.9 - 84.0 | 56.1 | 46.1 - 65.7 | 81.4 | 75.5 - 86.4 |
| >2000 | 56.00 | 45.7 - 65.9 | 81.98 | 76.3 - 86.8 | 58.3 | 47.8 - 68.3 | 80.5 | 74.8 - 85.5 |
| >2102 | 55.00 | 44.7 - 65.0 | 83.33 | 77.8 - 88.0 | 59.8 | 49.0 - 69.9 | 80.4 | 74.7 - 85.4 |
| >2200 | 55.00 | 44.7 - 65.0 | 84.23 | 78.8 - 88.8 | 61.1 | 50.3 - 71.2 | 80.6 | 74.9 - 85.5 |
| >2300 | 54.00 | 43.7 - 64.0 | 85.59 | 80.3 - 89.9 | 62.8 | 51.7 - 73.0 | 80.5 | 74.9 - 85.4 |
| >2400 | 53.00 | 42.8 - 63.1 | 87.39 | 82.3 - 91.5 | 65.4 | 54.0 - 75.7 | 80.5 | 74.9 - 85.3 |
| >2500 | 50.00 | 39.8 - 60.2 | 89.19 | 84.3 - 92.9 | 67.6 | 55.7 - 78.0 | 79.8 | 74.3 - 84.6 |

The best cutoff point (ng/mL) based on empirical cutoff-points estimation was (Youden method) >1265.

 **Supplementary Figure 2.** ROC curve of ferritin test for screening moderate-to-severe liver hemosiderosis (n= 400). Area under the ROC curve (AUC) 0.81 (95% CI 0.76 – 0.85), P<0.001.
